# Increased effect of two-fraction radiotherapy in conjunction with IDO1 inhibition in experimental glioblastoma

**DOI:** 10.1371/journal.pone.0233617

**Published:** 2020-05-29

**Authors:** Jonatan Ahlstedt, Elise Konradsson, Crister Ceberg, Henrietta Nittby Redebrandt

**Affiliations:** 1 Division of Neurosurgery, Department of Clinical Sciences, The Rausing Laboratory, Lund University, Lund, Sweden; 2 Department of Clinical Sciences, Medical Radiation Physics, Lund University, Lund, Sweden; Sechenov First Medical University, RUSSIAN FEDERATION

## Abstract

**Objectives:**

The aim of the study was to investigate therapeutic efficacy of single- or two-fraction radiotherapy in conjunction with IDO1-inhibition in a syngeneic rat glioblastoma model. IDO is known to cause immunosuppression through breakdown of tryptophan in the tumor microenvironment.

**Methods:**

Gene expression analyses of IDO in glioblastoma were performed with data from publicly available datasets. Fractionation studies were done on animals to evaluate tumor size, immune cell infiltration of tumors and serum profile on day 18 after tumor inoculation. Survival analyses were done with animals carrying intracranial glioblastomas comparing two-fraction radiotherapy+IDO1-inhibition to controls. IDO inhibition was achieved by administration of 1-methyl tryptophan (1-MT), and radiotherapy (RT) was delivered in doses of 8Gy.

**Results:**

The expression of IDO1 was increased on gene level in glioblastoma stem cells. Tumor size was significantly reduced in animals treated with 1-MT+RTx 2 (both long and short intervals, i.e. 7 and 4 days between the treatments) as compared to control animals, animals treated with only 1-MT or animals treated with 1-MT+RTx1. Serum levels of IL-1A were significantly altered in all treated animals as compared to control animals. Survival was significantly increased in the animals treated with 1-MT+RTx2 (7-day interval) compared to control animals.

**Conclusions:**

Addition of two-fraction RT to IDO1 inhibition with 1-MT significantly reduced tumor size in animals with glioblastoma. Survival was significantly increased in animals treated with two-fractioned RT+1-MT as compared to untreated controls increased significantly.

**Advances in knowledge:**

The currently used combination of only two fractions of radiotherapy and immune therapy is a promising area of research, increasing efficacy compared to single fraction irradiation, while potentially lowering radiation side effects compared to radiation in current clinical practice.

## Introduction

Immunotherapy has received much attention as a promising treatment for cancer of different types, and there is evidence of long-term therapeutic effects for some diagnoses. For glioblastomas, the most common primary malignant brain tumor, several clinical trials with different forms of immunotherapy are, or have been, conducted, unfortunately with lack of convincing clinical impact [1]. Current research is mainly focused on immune checkpoint inhibitors aiming at counteracting the tumor-associated suppression of the immune system, or various vaccines aiming at stimulating the immunological anti-tumor response [1]. However, many patients do not respond to immunotherapy, and the treatment-related toxicity can sometimes be severe when efforts are made to increase doses to potentially therapeutic levels, as seen in other forms of cancer [2, 3]. It is therefore relevant to consider alternative strategies, where doses could be reduced by efficient combinations, optimally resulting in synergistic effects.

More than half of all cancer patients receive radiotherapy, and it is estimated to contribute to about 40% of all cancer cures world-wide. It is also part of the current standard of care for glioblastoma. Radiotherapy aims for local tumor control, but it is now well established that radiation also has immunomodulatory effects, which can be explored in combination with immunotherapy [4, 5]. For many cancer diagnoses, clinical trials on the combination of immunotherapy and radiotherapy are already well under way [6]. Promising results have also been reported for malignant gliomas in animal models, both by us [7, 8] and by others [9–11]. The immunomodulatory effects of radiation are very complex, potentially evoking both stimulatory and inhibitory actions on the immunological anti-tumor response [12–17].

In the clinical setting, the established treatment protocol of astrocytoma WHO grade IV includes surgery with maximal tumor removal, followed by radiotherapy and concomitant as well as adjuvant temozolomide, nowadays also with the possibility to add tumor-treating fields to the temozolomide maintenance therapy [18]. Radiation therapy for glioma in a clinical setting is often fractioned as 2 Gy daily over 30 fractions administered five days per week. Less clinical experience is available regarding fewer or even single-fractioned radiation therapy [19], as well as its effects when combined with immunotherapy in this particular setting.

Indoleamine-2,3-dioxygenase (IDO) is a tryptophan catabolizing enzyme initially described as an immunomodulatory enzyme in the foeto-placental interface [20]. IDO is an inducible, intracellular enzyme, which is known to cause immunosuppression through breakdown of tryptophan in the tumor microenvironment [21]. Its’ potential for targeting in immune therapy has been explored on many cancer forms, but preclinical and clinical studies have failed to show efficacy in GBM when IDO inhibitors are used as monotherapy [22, 23]. Response has been observed in some patients when IDO inhibition has been added to standard of care on small phase 1b/phase 2 clinical studies [24], but the results are yet limited to the small sample size included in the studies and should of course be interpreted with care.

In previous studies, we have used a syngeneic ENU-induced rat glioma model (RG2) to investigate synergistic effects of hypo-fractionated radiation therapy in conjunction with IDO-inhibitory 1-DL-Methyl tryptophan (1-MT) treatment for GBM. We found increased survival in animals treated with one fraction of radiotherapy in conjunction to immune therapy with 1-MT [8]. The median survival time in rats treated with immunotherapy and a single radiotherapy fraction of 8 Gy at day 7 was 29 days (29±0.75). For the untreated controls, immunotherapy-only, and radiotherapy-only groups, the median survival time was 20±0.99 days, 18±0.28 days and 17±2.74 days respectively. We have applied a mathematical model developed by Serre et al. [25] to describe the combined effect of radiotherapy and immunotherapy [26]. Extrapolating from this provided that, in a two-fraction irradiation scheme, the synergy may be improved by short fraction intervals of 2–3 days, and that it decreased with increasing intervals. In this study, we attempt to investigate the therapeutic and immunological effects of irradiation of two treatment fractions in a novel and invasive rat glioblastoma model, in order to find ways to optimize the treatment while avoiding toxicity.

## Materials and methods

### Gene expression analysis

Datasets of Affymetrix gene expression array data was gathered from publicly available datasets using the NCBI Gene Expression Omnibus [27]. We collected sets where grade IV astrocytoma tissue samples were compared to biopsies from non-tumor brain gathered during epilepsy surgery. Log2-transformed fold change (Log2FC) in IDO-1 gene expression was compared between groups in each set.

### Rat glioma cells

The rat glioma cell line NS1 [28] was used. NS1 is a new GFP (green fluorescent protein) positive tumor cell line which was created by ENU treatment of pregnant homozygous GFP-positive Fischer 344 rats, where the offspring developed GFP-positive CNS-tumors, resulting in the NS1 cell line. Rats inoculated with NS1 cells develop cell-rich tumors with an invasive growth pattern. The tumors are positive for GFAP, GFP and the tumor cells have been shown to have a strong RNA expression for wt IDH1, wt p53, IDO1 and EGFR.

### Cell culture

The rat glioma cells (NS1) were cultured using RPMI-1640 (Sigma-Aldrich) medium with addition of 1% ml Na-pyruvate, 1% ml HEPES (4-(2-hydroxyethyl)-1-piperazineethanesulfonic acid), 0.1% ml gentamycin, as well as 10% inactivated fetal calf serum (heated to 56°C for 30 minutes). After culturing in T25 flasks, the cells were prepared for inoculation by removal of the medium and washed gently with PBS. Trypsin (Invitrogen) was added and cells were incubated in 37°C for 1–2 minutes to detach the adherent cells from the flask. More medium was added, and viable cells were counted. The cells were centrifuged at 1200 rpm for 5 minutes at 4°C, then the supernatant was carefully removed to avoid any potentially immunogenic calf serum. Afterwards the cell pellet was resuspended in serum-free medium (R0) to achieve a concentration of 1000 cells/μl.

### In vivo experiments

A total of 32 Fischer 344 rats were used in this study (Fischer Scientific, Germany). These rats were housed in pairs in rat cages with access to water and fed ad libitum with rat chow. The animals were monitored daily, and those displaying signs of paresis, epilepsy, or declined general condition were euthanized using carbon dioxide inhalation. If an animal should survive for 100 days without displaying symptoms, it would be euthanized on the same day in accordance with the ethical permission. No animals perished before meeting these endpoint criteria. This study was approved by the animal ethics committee in Lund with permit ID M15-16. All efforts were made to minimize animal suffering and all researchers in contact with the animals have undergone training in laboratory animal science at Lund University.

Each rat received an intracranial inoculation of 5000 cells from the GFP tumor line, suspended in 5 μl of nutrient solution. Intracerebral tumor cell inoculation was done under isoflurane inhalation anaesthesia using a stereotactic frame and a 10 μl Hamilton syringe. The cells were injected at a depth of 5 mm, 2 mm laterally from the sagittal suture, and at the coronary suture, on the right side of the cranium. The cranial burr hole was sealed with bone wax, and the incision was closed with absorbable suture.

### 1-Methyl tryptophan

In the 1-MT treated groups, a daily dose of 10 mg of 1-MT at a concentration of 4 mg/ml was administered via intraperitoneal injection under isoflurane anaesthesia, for 10 days starting at day 7 post inoculation (a similar dose to that used by us in [8]). 1-DL-MT was prepared by dissolving 4 g 1-DL-MT in 1 L 0.1 mol/L NaOH solution, after which pH of the solution was adjusted to 7.5 by adding HCl. Common dosage in clinical trials of indoximod have been 1200 mg administered twice daily [29]. This equates to approximately 35 to 40 mg/kg daily. For a Fischer rat of 150 g body weight, 10 mg daily is approximately 65 mg/kg daily.

### Radiation therapy

Radiation was administered in fractions of 8 Gy using a Xstrahl orthovoltage x-ray radiotherapy unit with beam energy set at 200 kV, with a 0.5 mm Cu filter. An applicator with a maximum field size of 40 mm x 40 mm was used, and the radiation field was further collimated to cover the frontal half of the brain (approximately 8 mm x 11 mm). The source-to-skin distance was 50 cm, and the absorbed dose rate was 1.2 Gy/min. During treatment, the animals were anesthetized by intraperitoneal injection of 60 mg/kg of ketamine. In order to estimate the resulting dose distribution to exposed normal tissues, the treatment set-up was reconstructed for a healthy rat in the microRayStation treatment planning system (RaySearch, Sweden) using a generic 200 kV x-ray unit. Organs-at-risk structures representing the cerebrum as a whole and the hippocampi specifically, were delineated.

### Fractionation study

Tumors were inoculated as described above. The animals were divided into five groups. One group consisted of untreated as controls (Group 1 = controls), and one received 1-MT treatment only (Group 2 = 1-MT).

Three treatment groups were given intraperitoneal 1-MT as well as irradiation to the inoculation site, either one fraction at day 7 post inoculation (Group 3 = 1-MT + RT 8 Gy x1), one fraction on days 7 and 11 (Group 4 = 1-MT + RT 8 GY x 2 short interval) or one fraction on day 7 and one on day 14 (Group 5 = 1-MT + RT 8 Gy x 2 long interval). All animals were sacrificed on day 18. The brains were analyzed for tumor size, estimating the area where the tumor was largest on coronal sections, assuming the tumor had the approximate form of an ellipsoid (*π*×*a*×*b*; where A = 2a and B = 2b are the lengths of the major and minor diameters).

To further analyze the effect of the fractionation interval, a mathematical tumor growth model described in a previous paper [26] was fitted to the tumor size measurements on day 18. In brief, the model accounts for the radiation-triggered recruitment and activation of immune effector cells, in synergy with the inhibitory effect of 1-MT on the tumor’s immuno-suppressive properties. In this work, the exponential tumor growth function of the original model was replaced by a Gompertz function, as this was subsequently found to better represent the intracranial growth of the present rat glioma model [30]. The model parameters were found by using the least square method with the lsqnonlin function in Matlab, whereas initial value estimates were obtained from our previous reports [26]. In order to estimate the uncertainty of the model, the fit was repeated 10,000 times with the data for each experimental treatment group resampled using bootstrapping with replacement.

### Survival study

In one animal trial, rats with intracranial NS1 gliomas were treated with 2 fractions of 8 Gy radiation to the inoculation site, in conjunction with intraperitoneal administration of 10 mg 1-MT daily for 10 days, with start at day 7 post inoculation (n = 6). Radiation therapy fractions were separated by one week apart. Tumor cell inoculation was made intracranially as described above, and radiotherapy and 1-MT were administered as described above. These animals were compared to untreated controls, who received tumor cell inoculations intracranially as described above, but no treatment (n = 6).

### Histology and serum analysis

At time of euthanization, blood samples were gathered by aspiration from the left ventricle of the heart, after which the animal was perfusion fixed using 4% paraformaldehyde solution. After fixation, brain tumor samples were collected. Tumor samples were homogenized in NP-40 serum with protein extraction buffer, at a concentration of 0.1 μg per μl.

Blood serum and homogenized tumor samples were analyzed using Bio-Plex 200 analysis (Multiplex immunoassays), a Luminex Technology based on flow cytometry immunoassays. Using the Bioplex technique, expressions of IL-1A, IL-1B, IL-2, IL-4, IL-5, IL-6, IL-10, IL-12, IL-13, GM-CSF, Ifn-G and TNF-A were analyzed.

Histological sections were made using paraffin infiltration protocols and all slides shown in this report have a thickness of 7 μm. Immunohistochemistry slides were performed using DAB staining kit. We compared tumor morphology and performed immunohistochemistry for CD4 (Sigma-Aldrich®), CD8 (Antibodies online®), and FOXP3 (Antibodies online®) with a hematoxylin counterstain, as well as hematoxylin-eosin staining for evaluation of tumor size.

### Statistical analysis

Statistical analysis as well as analysis of gene expression datasets was performed using R 3.4.0 within RStudio 1.0.143. All gene expression values were log2-transformed. Calculation of group mean differences was performed using t-test without assuming equal variance. Gene expression alteration were considered to be statistically significant for fold change > 2 and p-value < 0.05.

SPSS version 25 was used to compare Multiplex results and analyses if tumor size, using one-way ANOVA and considering p-values < 0.05 as statistically significant. Post-hoc Bonferroni corrected analyses were used to compare differences between the different treatment groups, considering p-values < 0.05 as statistically significant.

## Results

### Increased expression of IDO1 on gene level in two out of three datasets

Two large data sets showing gene expression PCR data on glioblastoma and normal brain tissue biopsies were analyzed (GEO accession ID:s GSE10878 [31], GSE4290 [32]). In the GSE4290 data set the Affymetrix Human Genome U133 Plus 2.0 Array platform was used to investigate global gene expression levels in 23 non-tumor brain samples and 77 samples from patients with glioblastoma. In the GSE10878 data set the Agilent-013282 Human Genome CGH Microarray 44B platform was used to compare gene expression in 4 non-tumor samples and 19 samples from patients with glioblastoma. In one of the GSE10878 data set, IDO1 overexpression was seen in tumor tissue compared to normal brain (Log2FC = 0.88, p = 0.024), but fold change >2 was not reached. In the largest of the datasets, GSE4290, no significant difference in expression was found (Log2FC = 0.049, p = 0.8).

**Fig 1:**
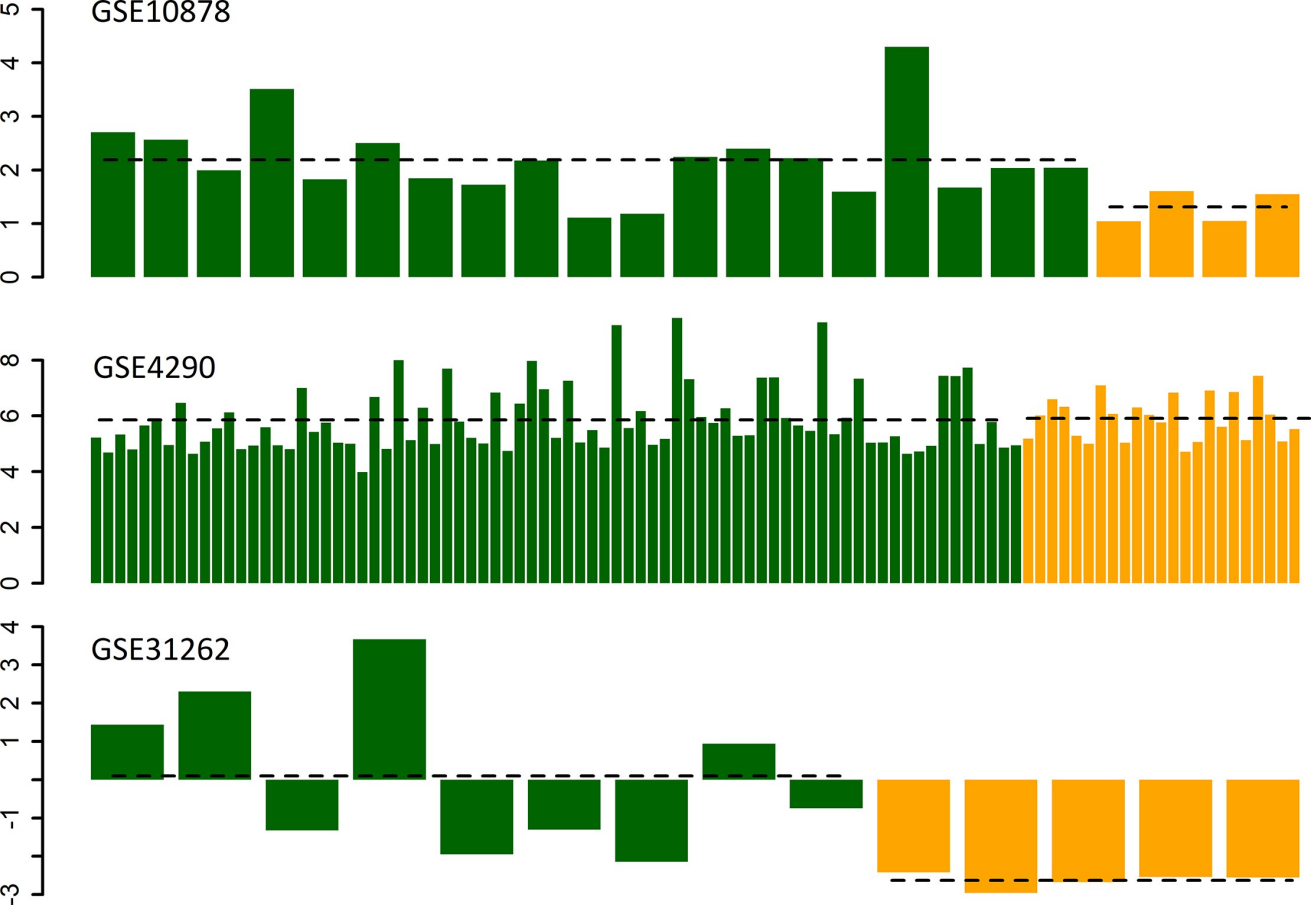
Gene expression of IDO1 in glioblastoma tissue (green) as compared to non-tumor tissue (yellow) with data analyzed from publicly available datasets. Group average expression in the glioblastoma groups and the control groups respectively is represented with the horizontal, dashed black line. On the y-axis log2-transformed fold change is represented, and on the x-axes the expression of IDO1 in the individual samples analyzed is presented. In the GSE4290 data set the Affymetrix Human Genome U133 Plus 2.0 Array platform was used to investigate global gene expression levels in 23 non-tumor brain samples and 77 samples from patients with glioblastoma. In the GSE10878 data set the Agilent-013282 Human Genome CGH Microarray 44B platform was used to compare gene expression in 4 non-tumor samples and 19 samples from patients with glioblastoma. In the GSE31262 data set the ABI Human Genome Survey Microarray Version 2 platform was used to compare gene expression between 5 individual samples of adult neural stem cells and 9 individual samples of glioma stem cells.

We also analyzed IDO1 expression in a data set where glioma stem cells and adult neural stem cells were compared (GEO accession ID GSE31262 [33]). In the GSE31262 data set the ABI Human Genome Survey Microarray Version 2 platform was used to compare gene expression between 5 individual samples of adult neural stem cells and 9 individual samples of glioma stem cells. Glioma stem cells showed a markedly increased IDO1 expression (Log2FC = 2.73, p = 0.005). This data is presented in Fig 1. Taken together this means that the most significant alteration of IDO1 expression was seen in glioma stem cells, with a fold change > 2; as compared to the other samples, where the fold change was < 2.

### Reduced tumor size in the irradiated animals as a function of dose and 1-MT treatment

**Fig 2:**
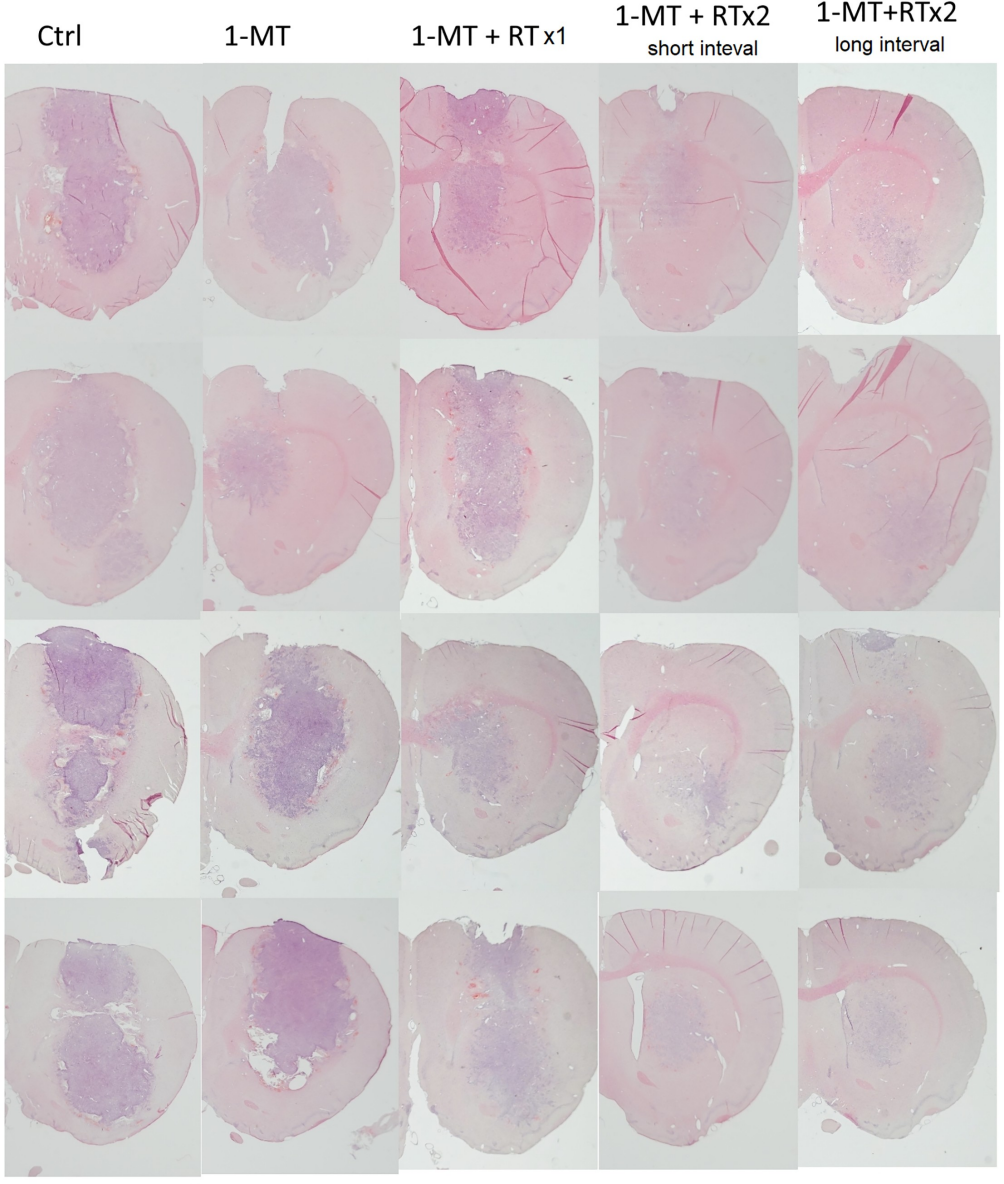
Coronal sections of the right hemisphere of the brains in animals with five different treatment regimen sacrificed 18 days after tumor cell inoculation and stained with hematoxylin(htx)-eosin are shown. In total the brains from 20 animals are seen, 4 in each treatment group. Infiltrative tumors were seen in both control animals and treated animals, and the characteristics of the tumors did not differ apparently between the groups with the regards to this. In the control animals, expansive tumors are seen, with densly packed glioblastoma cells, and tumor cell infiltration at the tumor border into the brain parenchyma. Furthermore, in animals treated only with 1-MT or RTx1+1-MT, large tumors are seen. In animals treated with 1-MT+RTx 2 (long and short interval), tumors are considerably smaller. The htx staining still demonstrate tumor cells, but not with a large tumor bulk with expansive effect, as seen in the other groups.

**Fig 3:**
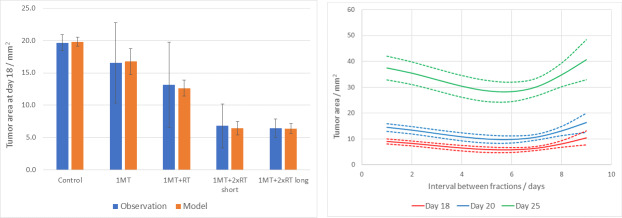
A) Mean tumor size upon euthanasia in the five treatment groups, shown by blue bars. Tumor size was significantly decreased in the animals treated with 1-MT+radiotherapy x 2. Orange bars represent model-fitted values. Y-axes represents the tumor area in mm^2^. Error bars indicate 1SD. B) Curves representing the cross-sectional area at selected time periods post inoculation as generated by the fitted model. Dashed lines indicate 1SD.

Twenty animals were divided into five groups as described in the methods section (n = 20 animals in total). Histology sections as well as statistics showing the tumor size as measured by the coronal cross section area are shown in Figs 2 and 3. There was an apparent reduction of tumor growth in rats as radiation dose went up as seen on the sections (Fig 2). Significant decreases in tumor size were seen between groups (ANOVA p-value 0.022), and Bonferroni corrected post-hos analyses showed a significant difference between animals treated with two fractions of radiotherapy plus 1-MT as compared to control animals (p = 0.009 RTx2 short interval+1-MT versus control; p = 0.007 RTx2 long interval+1-MT versus control). RTx1+1-MT or single 1-MT treatment did not differ significantly from the control group, and neither did the other between group analyses.

The goodness of the fit of the mathematical model is illustrated by Fig 3A, where the results of the model are shown together with the experimental data for the different treatment scenarios. The fitted model was then used to simulate treatment with 1-MT in combination with radiotherapy with two fractions and varying fractionation intervals, and the resulting tumor size at different time points are shown in Fig 3B. These results indicate an optimal fractionation interval of around 5 to 6 days, although the differences are small.

**Fig 4:**
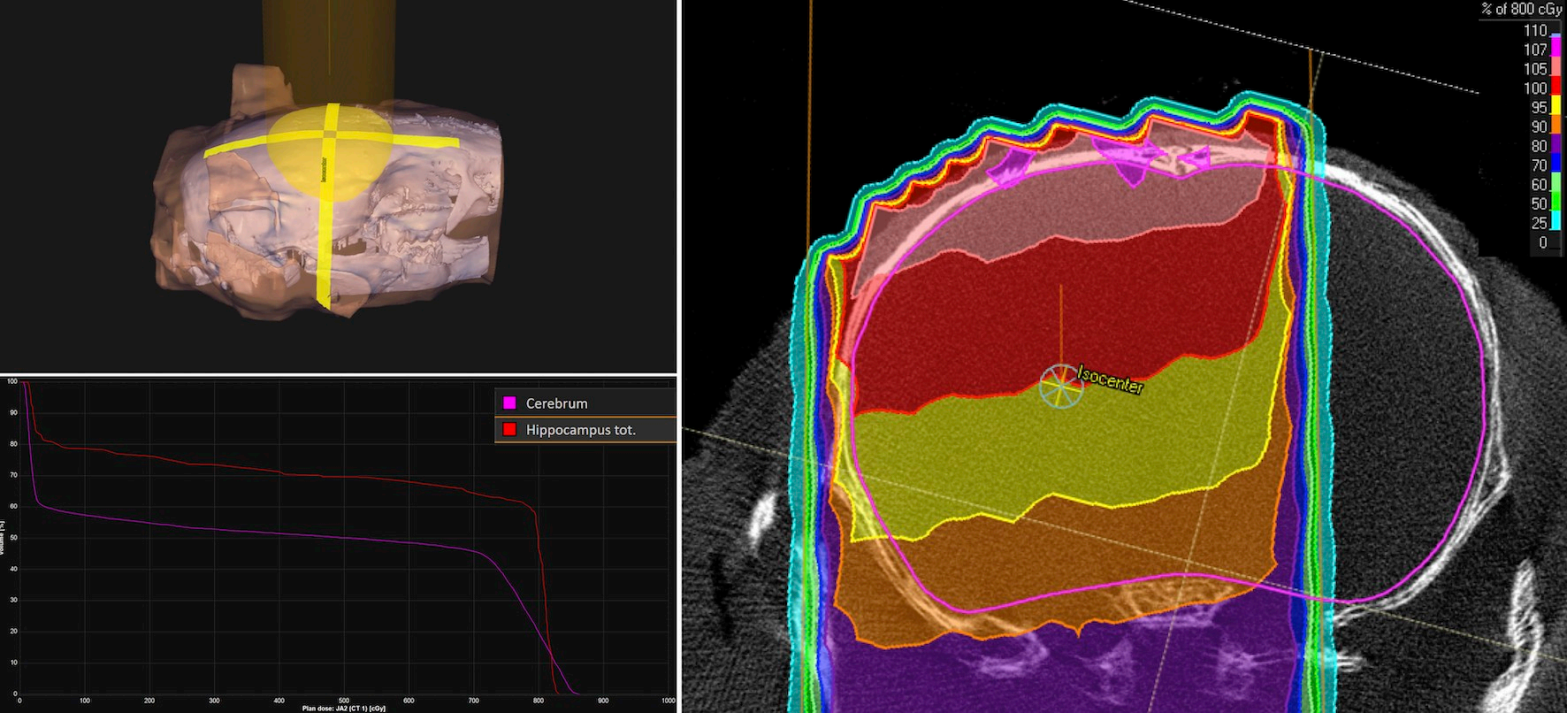
The reconstructed treatment set-up in the microRayStation treatment planning system, with the isocenter positioned at the point of the tumor cell inoculation. The upper left panel shows the orientation of the posterior radiation beam (10 mm field size) relative to a 3D rendering of the rat skull, with a semi-transparent skin contour and the cranium in white. The right panel shows the resulting dose distribution in an axial slice through the isocenter. The color shades illustrate the percentage dose levels relative to the 8 Gy prescribed dose to the isocenter. The lower left panel shows the dose-volume histograms for the cerebrum and the hippocampi.

The result of the reconstructed treatment set-up in the microRayStation treatment planning system shows that high dose levels extended into a large portion of the healthy cerebrum surrounding the tumor. In particular, about 60% of the volume of the hippocampi received full dose. About 40% of the cerebrum and about 20% of the hippocampi were spared almost completely. A reconstruction of dose distribution can be seen in Fig 4.

**Fig 5:**
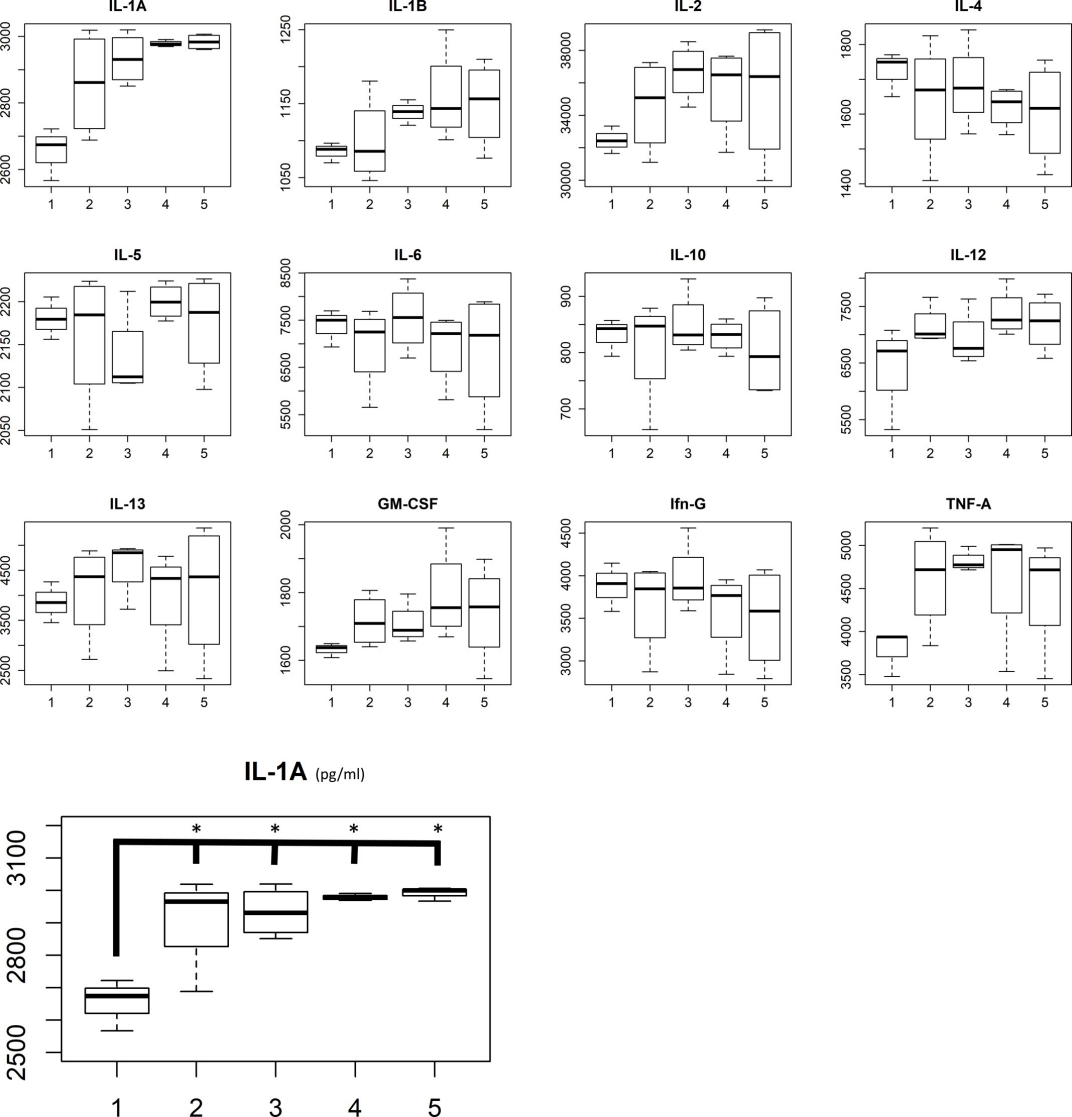
A) Multiplex analyses and B) IL-1A expression in the different treatment groups. Group 1 = control; 2 = 1-MT; 3 = 1-MT + RT x 1; 4 = 1-MT+RTx2 with short interval; 5 = 1-MT+RTx2 with long interval. On the y-axis concentration units are represented in pg/ml.

Serum cytokine concentration results are displayed in Fig 5A. One-way ANOVA was used to compare means across groups. IL-1A was significantly altered between groups (p = 0.006), whereas the other markers did not differ significantly between groups. Post-hoc Bonferroni corrected analyses was done on the IL-1A expression, with significant differences in expression between control group and all treated groups, but not between the different treatments (Fig 5B). For tumor tissue homogenates, values were out of range in the multiplex analysis, making further analysis impossible.

**Fig 6:**
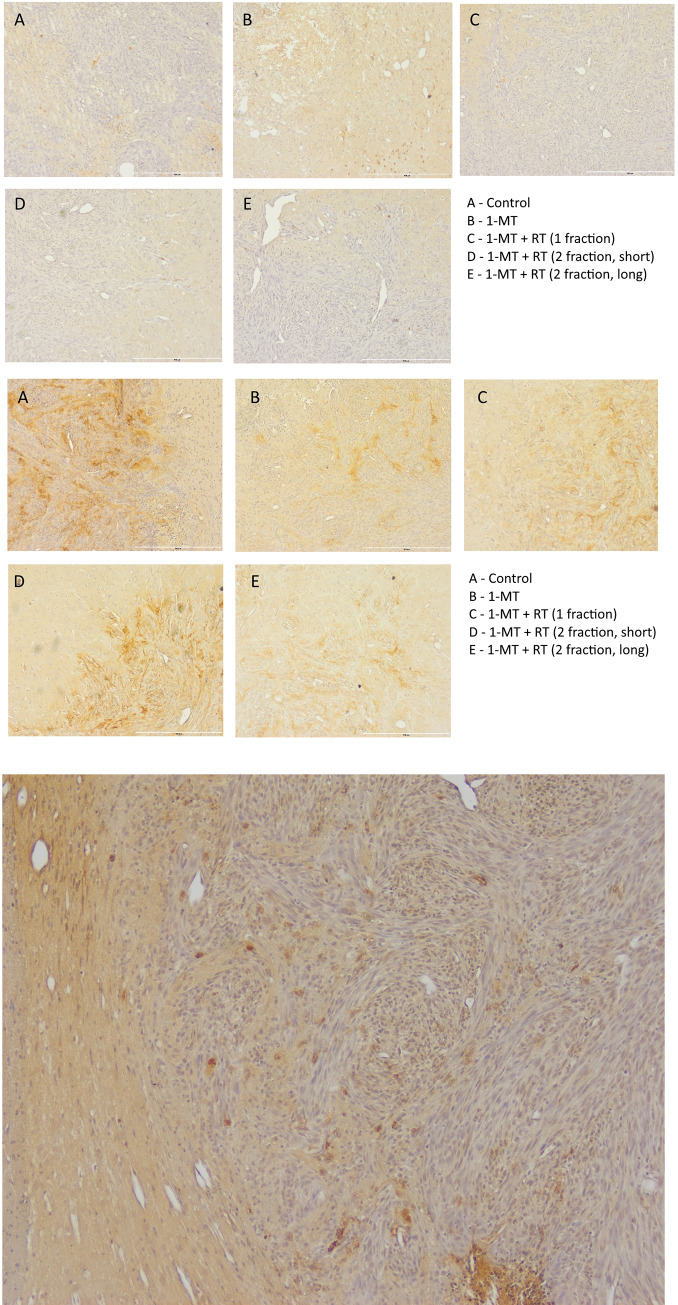
Htx stain with A) CD4 counter staining and B) FOXp3 counter staining on representative brains from each treatment group. C) Example image of CD8 stain.

In IHC stains with anti-CD4 antibodies, no apparent difference in CD4-positive cell numbers was observed in samples from different groups. A small number of CD4 positive cells could be observed infiltrating the tumor mass in all samples, as well as in adjacent brain tissue (Fig 6A). In FOXp3 stains, there was more widespread signal throughout the tumor mass, and not in surrounding tissue, suggesting a presence of regulatory T-cells (Fig 6B). A more intense FOXP3 staining was observed in control animals as compared to treated animals. CD8 positive cells are seen at the tumor periphery and around necrotic areas of tumors as exemplified in Fig 6C, again no apparent difference in cell numbers was observed upon visual inspection.

### Increased survival when combining 1-MT with two-fractioned radiotherapy (7 days between the two fractions)

**Fig 7:**
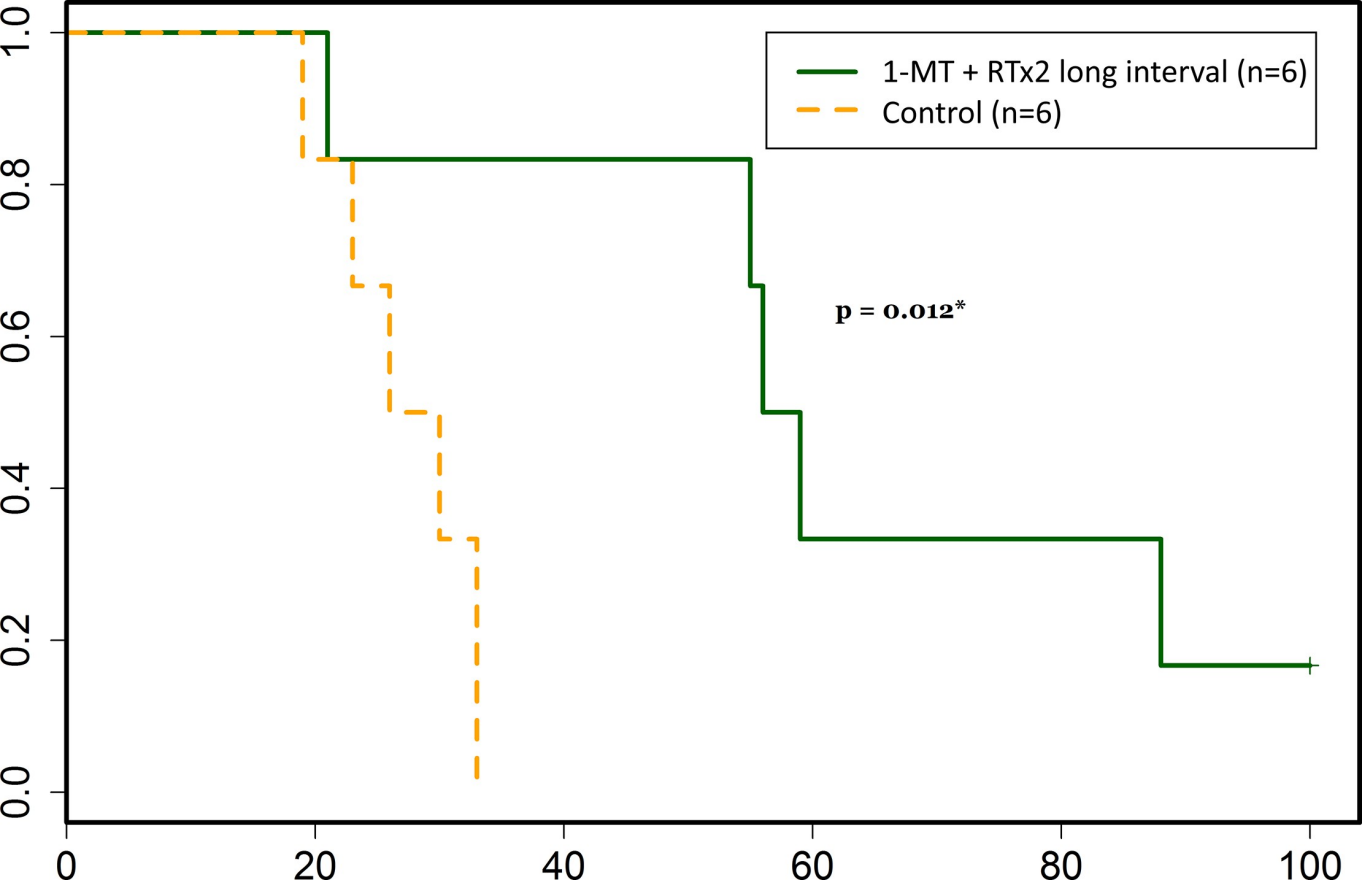
Survival was significantly increased in animals treated with 1-MT + RT x 2 (7 days between the two fractions) as compared to control animals (p = 0.012), which is demonstrated in the Kaplan-Meier curve presented here. Survival in days after tumor cell inoculation is represented on the x-axis, and the proportion of animals still alive is represented on the y-axis.

**Fig 8:**
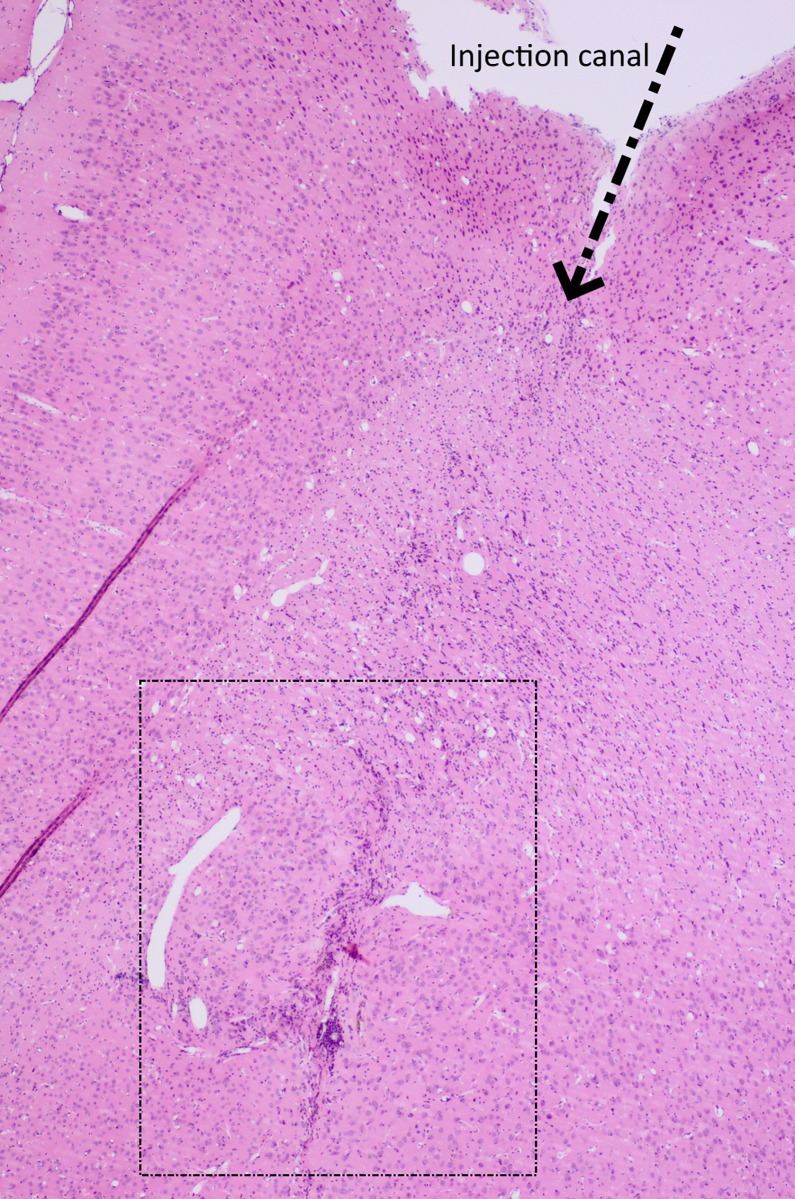
Htx and eosin stained section of the tumor site of one animal surviving for 100 days after tumor inoculation. The animal was treated with 1-MT and 2 fractions of 8 Gy irradiation separated by 7 days.

Survival in rats treated with a combination of 1-MT and two-fractioned radiation therapy with 8 Gy x 2 and 7 days between the two fractions (n = 6) compared with controls (n = 6) is detailed in Fig 7. Median survival was significantly longer in treated animals when compared to controls (63.2 ± 27.9 days vs 27.3 ± 5.7 days, p = 0.012). No side effects of the treatment could be observed as animals were examined daily regarding their general condition or any signs of neurological deficits. Histopathological sections showed some difference in tumor morphology, with control animals carrying the main portion of the tumor mass at the injection site while several treated animals seemed to have the main tumor mass located more superficially, along the injection needle path. This could theoretically be due to the fact that the treatment had effect on the tumor bulk at the time when it was delivered, early on in the course of the disease; and that the tumor cells that continued to grow were those in the margin of the tumor, causing a suspected recurrence later on. One of the treated animals was still alive at day 100 and was euthanized for ethical reasons at the termination date of the study. Histopathological changes in direct connection to the tumor inoculation path were found, but no sure tumor mass. This is shown in Fig 8. In one treated animal that perished at day 21, no tumor could be identified at the injection site. All control animals were found to carry tumors upon histopathological examination.

## Discussion

In the present study, we continued to explore the effects of combining single-fractioned or two-fractioned radiotherapy and immune therapy in an experimental intracranial glioblastoma model, finding a greater effect on tumor growth using two-fraction treatment compared to a single fraction, as well as a clear survival advantage in two-fractioned radiotherapy in 1-MT treated animals compared to untreated controls.

The net anti-tumoral effect of radiotherapy depends on the time, dose and fractionation of the radiation treatment [34, 35]. It has been argued that a single fraction of radiation would be preferable, since the recruitment of radiation sensitive immune cells could otherwise be counteracted by following treatment fractions. This could also allow for retreatment at later stages. However, there are also arguments in favor of multiple fractions [34]. Hence, in combination with immunotherapy, there is an opportunity to optimize the radiation treatment schedule to maximize the cooperative therapeutic effect.

The comparison between two-fraction and single-fraction radiation confirms a positive dose-response relationship regarding tumor growth. The question arises whether the reduction of tumor growth is strictly a cytotoxic effect of the radiation, or if part of the antitumor response is secondary to immunological consequences of the radiation. The presented model-generated predictions indicated that shorter intervals would be more beneficial than longer intervals, whereas the results obtained from the present NS1 model failed to strengthen this hypothesis. Discrepancies between the predictions and the observations may be explained by the fact that the different tumor cell lines behave differently in this setting, as the base data for the predictions was gathered using the RG2 glioma model. The RG2 glioma model is now considerably dated, and consideration should be taken to the fact that long-term and repeated tumor cell culturing could have caused the model to lose resemblance to human primary glioma. This risk is reduced by using a novel but stable cell line like NS1, which is one of its reasons of conception.

We characterized the antitumor response by mapping infiltration of CD4+ and FOXp3+ cells in the tumor microenvironment. The results show the FOXp3 signal in the tumor mass as strong in the control animals, which may indicate a strong immunosuppressive Treg effect, which may be mitigated by administering the combined treatment.

The increase of IL-1A seen in serum analyses would seem predictable considering its role as an inflammatory marker, as radiation predictably causes inflammation. Recently, microdialysis has been used to measure some IL expressions in glioblastoma and adjacent brain tissue, showing that there was a positive correlation between baseline IL-8 and IL-6 microdialysis levels in tumor tissue and survival. Furthermore, a significant increase of IL-8, MCP-1 and MIP-1A were detected in tumor tissue already after the first dose of radiation and increased further during 5 days of radiation [36].

We chose to test the treatment schedule of two fractions with long intervals between the fractions in combination with 1-MT in the survival study and could demonstrate a significant survival advantage as compared to control animals, with no observable side effects. The survival was more dramatically improved with this protocol in the model tested here with the NS1 cell line, as compared to the results generated with RTx1 + 1-MT in our previous work with RG2 [8]. We hypothesize that, by administrating radiotherapy in only two fractions, we can increase its immunogenic effects. This could possibly explain the difference in relation to results presented in other models, for example by Ladomersky et al. [22], who could see effect of radiotherapy and IDO inhibition only when combined with anti-PD-1 antibodies as well. In the study by Ladomersky et al. [22], five fractions of radiotherapy were administered on five consecutive days, another IDO inhibitor was used and the experiments were done in mice, meaning that direct comparisons should be interpreted with care.

The gene analyses of IDO1 expression in human glioblastoma revealed the interesting finding that IDO1 was overexpressed mainly when comparing glioblastoma stem cells to neural stem cells, and not in GBM tissue compared to normal brain tissue. Only in glioma stem cells, a significant increase with fold change > 2 was observed. This could indicate that IDO1-expression in certain sub-populations of GBM is an important driver of tumor progression and immune suppression in glioblastoma. It has also been shown by Ladomersky et al. [37] that IDO expression seems to be increased in brain samples of “normal” brain from older patients, which further could add to the hypothesis that immune suppression is increased in these population and possibly part of the explanation to the fact that age is such an important prognostic factor in patients with glioblastoma. However, it should be added that the increased IDO expression in so called normal brain was seen only in the age span 60–69 years, and not older or younger brains, which of course raises the question regarding generalizability of these data, and points to the need for further validation.

Novel IDO inhibitors have reached the market with the possible advantage of being more specific to and stronger inhibitors of IDO1 compared to 1-MT, which in its D-isomer (indoximod) binds to and inhibits primarily IDO2, whereas the L-isomer has a stronger affinity to and inhibition of IDO1. Indoximod has been demonstrated to have a stronger anti-tumor effect in some studies compared to its L-isomer even though it is a very weak inhibitor of IDO1. The mechanism by this is proposed to be the reactivation of mTORC1 activity by indoximod acting as a tryptophan substitute. Epacadostat in comparison selectively targets and binds to IDO1, reducing the catabolization of tryptophan in the tumor microenvironment. 1-DL-MT is still in widespread pre-clinical use, as its effects are that of reversing the effects of IDO1 activity, while being very well tolerated by research animals in therapeutic doses. Other new IDO1 inhibitors have emerged, such as BMS-985205, PF-06850003, and navoximod, being investigated in preclinical and clinical research for different tumor types [38, 39].

Manipulating RT fractioning and dosages to further enhance these immunological effects is an avenue of great interest to us. While RT dosage and fractioning equivalencies in small animals and humans are not well understood and more research is needed in this area, it would be interesting to combine the two-fractioned radiotherapy with novel IDO1-inhibitors as well as anti-PD-1 treatment in our animal model with fully immunocompetent rats and an infiltrative glioblastoma model, which has not been cultured in vitro for decades. Possible drawbacks with the more intense treatment suggested above include unwanted side effects.
